# Associated predictors of functional impairment among adolescents with ADHD—a cross-sectional study

**DOI:** 10.1186/s13034-022-00463-0

**Published:** 2022-04-05

**Authors:** Jenny Meyer, Iman Alaie, Mia Ramklint, Johan Isaksson

**Affiliations:** 1 Child and Adolescent Psychiatry Unit, Department of Medical Sciences, Uppsala University, 751 85 Uppsala, Uppsala, Sweden; 2 Division of Insurance Medicine, Department of Clinical Neuroscience, Karolinska Institutet, Stockholm, Sweden; 3 Centre for Psychiatry Research, Department of Women’s and Children’s Health, Karolinska Institute Centre of Neurodevelopmental Disorders (KIND), Karolinska Institutet, Stockholm, Sweden

**Keywords:** ADHD, Functional impairment, Adolescents, Multiple informants

## Abstract

**Background:**

Attention-deficit/hyperactivity disorder (ADHD) in adolescence is associated with functional impairment in several domains of life. To enable development of interventions that more effectively target functional impairment in this age group, the associations between clinical characteristics and impairment need to be clarified. The aim of this study was to investigate the associations between ADHD and functional impairment, if they varied by sex, and the potential impact of comorbid psychiatric symptoms on the associations.

**Methods:**

This was a cross-sectional study including adolescents with ADHD (n = 164) and a reference group of adolescents without ADHD (n = 106). Self-ratings and parental ratings of functional impairment in different life domains were used as outcomes in all analyses. Differences between groups were investigated with comparative analyses. General linear models (GLMs) were used to explore associations between ADHD symptoms and functional impairment in adolescents with ADHD, while adjusting for of comorbid symptoms, sex, and medication.

**Results:**

Adolescents with ADHD displayed higher levels of functional impairment than peers without ADHD, and girls with ADHD rated higher impairment than their male counterparts. The combined ADHD presentation was associated with the highest levels of self-reported impairment, while parental ratings indicated comparable levels of overall impairment across presentations. In the adjusted GLMs, symptoms of inattention were strongly associated with self- and parent-rated impairment in school, but symptoms of hyperactivity/impulsivity were not, whereas symptoms of both inattention and hyperactivity/impulsivity were modestly associated with self-rated impairment with friends. Further, both emotional and conduct problems were associated with impairment in daily life.

**Conclusions:**

Our results suggest that attention difficulties, in particular, seem to impair academic functioning in adolescents with ADHD, and interventions targeting such difficulties are warranted. In addition, comorbid symptoms need to be assessed and treated, and self-reports of functioning should be included in research and clinical practice involving adolescents.

**Supplementary Information:**

The online version contains supplementary material available at 10.1186/s13034-022-00463-0.

## Background

Attention-deficit/hyperactivity disorder (ADHD) is characterized by age-inappropriate and impairing symptoms of inattention and/or hyperactivity/impulsivity, and is related to functional impairment in several life domains [1], such as school [2, 3], social relationships [4, 5], and at home [6, 7]. ADHD is categorized into three main presentations: the combined presentation (ADHD-C), the predominantly inattentive presentation (ADHD-I), and the predominantly hyperactive/impulsive presentation (ADHD-H) [1]. However, there is heterogeneity within each ADHD presentation and individuals are likely to shift between different presentations over time [8, 9]. Previous research has found support for the validity of distinguishing between the symptom domains of inattention and hyperactivity/impulsivity, and a more dimensional perspective on ADHD has been suggested, where the symptom load in both these domains should be considered at each time of assessment [10, 11]. While overt symptoms of hyperactivity tend to decrease with age, symptoms of inattention and functional impairment often persists [1–3, 11–14]. In addition to the core symptoms, about two-thirds of adolescents with ADHD suffer from psychiatric comorbidities, such as oppositional defiant disorder (ODD), conduct disorder, major depression, and anxiety disorders [15, 16], which may exacerbate impairment in daily life.

### Functional impairment in adolescents with ADHD

The magnitude of academic impairment may become more pronounced in adolescence, since academic demands often increase in high school (e.g., more lectures, writing assignments, homework, exams) [17]. Many adolescents with ADHD also display impairment in social relationships [4, 5], more often being rejected by peers, engaging in risky behaviors, and showing difficulties in forming sustained high-quality relationships [18]. Moreover, ADHD is associated with elevated levels of conflicts and stress within the family [6, 7]. Since functional impairment is often the primary reason for referral to health care, improvement of daily functioning should be one of the main goals when treating adolescents with ADHD [19, 20]. However, evidence for treatment effects on functional impairment in specific life domains and the persistence of such effects over time is still scarce for this age group [20, 21]. In order to target functional impairment in adolescents with ADHD, it is crucial to learn more about how the core symptoms of ADHD and comorbid symptoms are related to functional impairment in different life domains.

### The influence of ADHD symptoms on functional impairment

Though ADHD symptoms and functional impairment are related, they are separate constructs. Accordingly, previous studies have shown small to moderate correlations between them [10] and a decrease of ADHD symptoms does not always correspond to a decrease in impairment [22–24]. The associations between ADHD symptoms and parent- and/or teacher-rated functional impairment have been explored from a few, slightly different perspectives [9–11]. In regard to ADHD presentations, Willcutt et al. found that all presentations were related to elevated levels of functional impairment as compared to neurotypical peers [10]. However, ADHD-C was associated with the lowest levels of overall functioning and social functioning, while ADHD-I predicted levels of academic impairment comparable to those with ADHD-C [10]. When using a more dimensional perspective on the diagnosis, inattention has been found to be the strongest associated predictor of impairment of academic functioning, whereas both inattention and hyperactivity/impulsivity have been associated with social impairment [10, 11]. However, the symptom domains seem to be related to different aspects of social impairment. Inattention appears to be related to a higher tendency to display passive social behavior, while hyperactivity/impulsivity has been associated with an increased risk of relational aggression and rejection by peers [10]. In a study by Gardner et al. [9], the associations between the ADHD domains and parent- and/or teacher-rated functional impairment were explored, with account taken of comorbid symptoms, sex, age, and medication status. The findings from this adjusted model confirmed inattention as the strongest predictor of academic impairment, whereas hyperactivity was found to be more strongly associated with disruptive behaviors in the classroom. When oppositional symptoms were accounted for, ADHD symptoms explained only minimal variance in interpersonal problems [9].

### The influence of comorbid symptoms on functional impairment

A majority of adolescents with ADHD struggle with symptoms of psychiatric comorbidity [15, 16]. A recent Swedish registry study [25] reported that children with ADHD and a psychiatric comorbidity had lower scores on global functioning than children who had ADHD only. Symptoms of ODD have been shown to be more strongly associated with concurrent interpersonal problems than symptoms of ADHD [9], and in a study of young adolescents with ADHD, both conduct problems and symptoms of depression were identified as risk factors of social impairment [5]. ADHD in combination with anxiety and affective comorbidity has recently been associated with an increased risk of impairment in academic performance [26]. In addition, longitudinal studies have identified psychiatric comorbidities as predictors of negative long-term outcomes of ADHD [13]. These findings highlight the need of considering comorbid symptoms when assessing functional impairment in youths with ADHD.

### The influence of sex on functional impairment

When investigating the associations between ADHD and functional impairment, potential sex differences might be important to consider. In a recent population-based study, parents rated boys as more impaired than girls [11], while other studies on children with ADHD generally showed comparable levels of parent-rated impairment across the sexes [27–29]. A previous meta-analysis indicated that some sex differences were mediated by referral source, where studies of non-referred children with ADHD found more impairment in boys, whereas studies of clinic-referred samples showed comparable levels across the sexes [28]. In contrast, some studies of young adults with ADHD have suggested that females perceive themselves as more impaired than their male counterparts [30–32]. Hence, the impact of sex on perceived impairment remains unclear and may to some extent be related to both the type of sample (e.g., population-based sample or clinical sample) and the informant (e.g., parent or a patient him-/herself).

### The adolescent perspective on functional impairment

Most studies investigating the association between clinical symptoms and functional impairment for youths with ADHD have been conducted on younger children or pooled samples of children and adolescents [9–11, 25, 26]. Moreover, an overwhelming proportion of these studies are based on ratings from other informants, such as parents, teachers, or clinicians [9–11, 25, 26], while self-ratings are more frequently used in adult populations [10, 30–33]. Adolescents often spend a large amount of time outside the family context [18], some distancing from parents usually takes place [34], and overt symptoms of ADHD tend to decrease [1, 10, 12]. Hence, both symptoms and daily impairment may be less observable for others and relying solely on other informants may therefore be problematic in studies of adolescents [5, 19]. Taken together, this means that adolescents’ own perspectives on the associations between ADHD symptoms and functional impairment in daily life have often been overlooked. Increased knowledge of how symptoms are related to self-reported impairment could move us one step closer to more effective and acceptable interventions for this age group.

### Aim and hypotheses

The aim of this study was to investigate how self- and parent-rated functional impairment related to ADHD and sex, and how the symptom domains of ADHD were associated with impairment in different life domains when adjusting for comorbid symptoms. We hypothesized:(i) that adolescents with ADHD would have higher ratings of functional impairment than peers without ADHD, (ii) that the highest levels of overall impairment would be seen for ADHD-C, while the levels of impairment in school would be about the same for ADHD-C and ADHD-I, (iii) that symptoms of inattention would explain most of the variance in impairment in school, while both symptom domains would be significantly associated with impairment with peers and at home, and (iv) that the explained variance of functional impairment resulting from each ADHD domain would be attenuated when adjusting for conduct problems and emotional problems, especially in regard to impairment with peers and at home.

## Methods

### Design, participants, and procedure

This was a cross-sectional study including a clinical sample of adolescents with ADHD (*n* = 164, mean age: 16.6 years (standard deviation (*SD*): 0.99), 64.0% female) and a community-based sample of adolescents without ADHD (*n* = 106, mean age: 16.6 years (*SD*: 1.02), 68.9% female), with the latter serving as a reference group. The clinical sample stemmed from our previous multi-center study, evaluating psychological group treatment for adolescents with ADHD [35, 36]. The participants were recruited via child and adolescent psychiatric (CAP) outpatient units in seven regions of Sweden between 2015 and 2018. The adolescents and their parents were informed about the study through written information in waiting rooms and through verbal and written information delivered by clinical staff. All participants were informed that they would be randomized to one of two group interventions, that participation involved responding to questionnaires, and that participation was voluntary. In the current study, we used data only from pre-treatment assessments. All the included CAP units had large catchment areas with patients from both rural and urban settings. The inclusion criteria were an ADHD diagnosis (retrieved from the patient’s medical record), being aged 15–18 years, and having completed pre-treatment measurements (self-reports and/or parental reports) on a digital platform from home. Exclusion criteria were severe depression, suicidality, psychosis, or bipolar disorder without stable medication, intellectual disability, organic brain injury, autism spectrum disorder, or current substance abuse. Assessment of study eligibility was performed by clinical psychologists, who interviewed the adolescents and their parents at the CAP units. The final clinical sample included 164 participants (159 self-reports and 162 parental reports).

The reference group was recruited during 2019 at local high schools in the region of Uppsala. A research assistant presented written and verbal information about the study. The students were informed that participation involved completion of questionnaires and that it was voluntary. Inclusion criteria were being aged 14–19 years and having responded (or had parents respond) to a set of questionnaires, completed on a digital platform from home. Exclusion criteria were an ADHD diagnosis, as reported by the parents, or clinical levels of ADHD symptoms (only applied in cases where parental ratings were missing (n = 12)). For participants for whom only self-ratings were available, the Adult ADHD self-report scale for adolescents (ASRS-A) [37] was used to determine clinical levels of ADHD symptoms, based on a dichotomized cut-off, where a cut-off score of 9 or higher was used [37, 38]. Eight adolescents were excluded from the reference group based on either a parental-reported ADHD diagnosis (n = 4) or clinical levels of self-reported ADHD symptoms (n = 4). In total, 106 participants were included in the reference group (103 self-reports and 94 parental reports).

### Measurements

#### ADHD diagnosis

ADHD diagnosis was used as a dichotomous variable to compare the clinical sample to the reference group. The variable was dichotomized as either having or not having ADHD.

#### Sex and age

For the clinical sample, each participant’s sex and age were retrieved from their personal identity number, whereas this information was based on self-reports for the reference group. Sex was used as a dichotomous variable (male or female) and age as a continuous variable.

#### Medication status

Ongoing medication for ADHD was reported by the parents in the clinical sample. Medication status was used as a dichotomous variable (ADHD medication or no ADHD medication).

#### Current ADHD presentation

Since the participants in the clinical sample were diagnosed with ADHD prior to the study (in some cases, several years earlier), all eligible participants were diagnostically reassessed. Clinical psychologists performed this reassessment at the CAP units, together with the adolescents and their parents, using the module for ADHD in the Mini International Neuropsychiatric Interview for Children and Adolescents (MINI-KID) [39]. The current presentation of ADHD symptoms was based on the number of prevalent symptoms in the preceding six months, and assessed in accordance with the fifth edition of the Diagnostic and Statistical Manual of Mental Disorders [1]. Participants who currently fulfilled fewer than six symptoms of both inattention and hyperactivity/impulsivity (< five symptoms for adolescents aged 17 years and older), were categorized as unspecified ADHD (ADHD UNS). Since a substantial proportion of the clinical sample was categorized as ADHD UNS (18.3%), we included this diagnosis as a category in the variable “current ADHD presentation.” As only three individuals were categorized as manifesting ADHD-H, it was deemed that they should be excluded from the analysis of ADHD presentation. Current ADHD presentation was used as a nominal variable: ADHD-C, ADHD-I, or ADHD UNS.

#### Symptom domains of ADHD

Symptoms of inattention and hyperactivity/impulsivity were assessed with self-ratings and parental ratings on two different subscales of the ASRS-A [37, 40]. This questionnaire contains nine items on each subscale, corresponding to the diagnostic symptoms of the two ADHD domains. The occurrence of each symptom is measured on a 5-point scale, ranging from 0 (never) to 4 (very often). The symptom domains were treated as continuous variables, using raw scores, with higher scores indicating higher symptom severity (min = 0, max = 36 for each subscale). Based on the clinical sample in this study, the subscale for inattention showed good internal consistency (*α* = 0.85 for self-ratings and *α* = 0.83 for parental ratings), as did the subscale for hyperactivity/impulsivity (*α* = 0.88 for both self-ratings and parental ratings).

#### Comorbid symptoms

Emotional problems and conduct problems were assessed with self-ratings and parental ratings on the Strengths & Difficulties Questionnaire (SDQ) [41–43]. The questionnaire encompasses five items about the occurrence of emotional problems (e.g., “I worry a lot” or “I am often unhappy, depressed or tearful”) and five items regarding the occurrence of conduct problems (e.g., “I fight a lot. I can make other people do what I want” or “I usually do as I am told” (reversed)). Each item is measured on a 3-point scale from 0 (not true) to 2 (certainly true), where higher scores indicate more severe problems (min = 0, max = 10 for each subscale). Based on the clinical sample in this study, Cronbach’s *α* for emotional problems was 0.71 for self-ratings and 0.66 for parental ratings, whereas Cronbach’s *α* for conduct problems was 0.61 for self-ratings and 0.62 for parental ratings, which indicated questionable to acceptable internal consistency for these subscales. Notably, the subscales of the SDQ should be used only to screen for emotional and behavioral difficulties, not for diagnostic purposes.

### Outcomes

Functional impairment was measured with self-ratings and parental ratings of the Child Sheehan Disability Scale (CSDS) [44, 45]. The self-rating scale assesses functional impairment in three areas (school, social activities with friends, and at home). The original parent version of the CSDS consists of five items, including two items assessing impairment in the parent’s daily activities (work, social life). However, in this study, we used only the three items that refer to impairment in the child’s daily life, which correspond to the same areas as the self-ratings. Notably, the phrasing and meaning of the question on impairment at home differs slightly between the self-rating scale and the parental rating scale. The self-rating question asks if the symptoms lead to any problems for the *adolescent* at home, while the parental rating question asks whether the child’s symptoms have any negative impact on their *joint family life*. Impairment in each area is measured on an 11-point scale from 0 (not at all) to 10 (very much), with higher scores indicating more impairment. In the current study, we investigated both overall impairment (full scale) and impairment in each area. The CSDS displayed good internal consistency (*α* = 0.82 for self-ratings and *α* = 0.89 for parental ratings) in this study.

### Power calculations

Power calculations were performed using G*power 3.1.9.6. For the group comparisons (*n* = 164 in the ADHD group, *n* = 106 in the reference group, power = 0.80, *α* = 0.05), the study was statistically powered to detect small to medium effects (*d* = 0.37). For comparing the outcomes between ADHD presentations (*n* = 164, power = 0.80, *α* = 0.05), the study was powered to detect medium effects (*f* = 0.25). In regard to the multiple regression model, including six potential predictors (*n* = 164, power 0.80, *α* = 0.05), the study was powered to detect small to medium effects (*f*^*2*^ = 0.09).

### Analyses

All analyses were conducted using IBM SPSS Statistics, version 28.0. Descriptive statistics were used to present the characteristics of the two groups. Sex differences in medication status within the clinical sample were explored with the chi-squared test. Due to skewness in the reference group, non-parametric statistics were used in all analyses involving this group. Spearman’s rho was used to explore the correlations between self- and parent-rated impairment. Group differences in sex distribution, age, psychiatric symptoms, and functional impairment in different life domains were examined using the chi-squared test for the dichotomous variable and Mann–Whitney U-tests for continuous variables. Potential differences across combinations of group and sex in regard to perceived functional impairment were explored with the Kruskal–Wallis test (with pairwise group comparisons).

As regards the clinical sample of adolescents with ADHD, the assumptions of normality, linearity, homoscedasticity, and the absence of multicollinearity were met. Mean differences in functional impairment (overall, school, friends, home) across ADHD presentations (ADHD-C, ADHD-I, and ADHD UNS) were investigated with one-way analysis of variance, with Tukey’s post-hoc test used to explore potential differences further. To investigate the variance explained by each ADHD domain (inattention and hyperactivity/impulsivity) on the outcomes of functional impairment in adolescents with ADHD, general linear models (GLMs) were conducted in two steps and investigated in separate models for each outcome. In the first step, inattention and hyperactivity/impulsivity were introduced into the model without any further adjustments, to observe their crude effects on the outcomes. Next, conduct problems, emotional problems, sex, and medication status were added to the model, to explore if the effect sizes of the ADHD domains were preserved and if the other independent variables contributed to the explained variance of the outcomes. Partial eta squared was used as a measure of effect size (0.01 = small, 0.059 = moderate, 0.138 = large). Results were considered significant at the 5% level.

## Results

### Sample characteristics

No differences were found between the two groups in regard to distribution of age (*U* = 8733.00, *p* = 0.95) or sex (*χ*^*2*^ = 0.672, *p* = 0.41). A majority of the participants in the clinical group were on pharmacological treatment for ADHD (76.5%), and ADHD medication was more common among male than female participants (86.4% vs 70.9%, *χ*^*2*^ = 5.063, *p* = 0.02). In regard to current ADHD presentation, ADHD-C was most common (44.5%), followed by ADHD-I (35.4%), ADHD UNS (18.3%), and ADHD-H (1.8%). Correlations between self- and parent-rated impairment were low to moderate for both the ADHD group (*ρ* = 0.22–0.42, *p* < 0.01) and the reference group (*ρ* = 0.33–0.46, *p* < 0.01).

### Group differences and the potential influence of sex

Descriptive statistics and group differences regarding psychiatric symptoms and functional impairment are presented in Table 1. The ADHD group displayed higher levels of self- and parent-rated functional impairment in all life domains, as compared with the reference group. In the pairwise comparisons investigating differences in functional impairment across combinations of group and sex, both adolescent boys and girls with ADHD were found to report significantly higher levels of overall impairment and impairment in school and at home, as compared with both sexes of the reference group. Girls with ADHD had higher ratings on overall impairment and impairment in school and with friends than boys with ADHD, whereas no sex differences were observed within the reference group. Boys with ADHD displayed levels of impairment with friends comparable to those of both boys and girls without ADHD. Child sex did not influence parent-rated impairment. Results from the comparative analyses across combinations of group and sex are displayed in Additional file 1: Table S1.

**Table 1:** Descriptive statistics (mean (SD)) and group differences regarding psychiatric symptoms and functional impairment

Characteristics	ADHD group (n = 164)			Reference group (n = 106)			Group differences, U
	All	Males	Females	All	Males	Females	
Inattention							
Self-rated Parent-rated	23.13 (6.78) 25.82 (5.59)	19.38 (6.71) 25.14 (4.89)	25.28 (5.84) 26.20 (5.94)	13.43 (5.73) 7.78 (6.02)	14.28 (5.77) 8.52 (5.82)	13.04 (5.71) 7.45 (6.12)	14,044.50*** 14,842.00***
Hyperactivity/impulsivity							
Self-rated Parent-rated	18.87 (8.04) 17.96 (7.65)	16.48 (7.33) 17.84 (6.18)	20.24 (8.14) 18.03 (8.41)	10.57 (6.04) 4.70 (4.88)	12.28 (5.89) 5.10 (5.21)	9.80 (5.98) 4.52 (4.76)	12,868.50*** 14,032.00***
Emotional problems							
Self-rated Parent-rated	4.80 (2.55) 4.46 (2.41)	3.30 (2.29) 3.25 (2.15)	5.65 (2.29) 5.15 (2.29)	4.11 (2.61) 1.63 (1.95)	3.38 (2.87) 1.28 (1.49)	4.44 (2.44) 1.78 (2.11)	9541.00* 12,537.00***
Conduct problems							
Self-rated Parent-rated	3.04 (1.98) 2.83 (2.00)	2.78 (2.05) 3.02 (2.27)	3.20 (1.92) 2.73 (1.83)	1.47 (1.35) 0.72 (1.07)	1.78 (1.62) 0.76 (1.21)	1.32 (1.19) 0.71 (1.01)	12,174.50*** 12,521.00***
Overall impairment							
Self-rated Parent-rated	14.24 (7.33) 17.48 (6.17)	11.79 (6.77) 17.13 (6.13)	15.64 (7.30) 17.69 (6.21)	6.91 (6.23) 2.07 (4.35)	7.19 (6.39) 1.17 (2.44)	6.79 (6.19) 2.48 (4.94)	12,760.50*** 14,719.00***
Impairment in school							
Self-rated Parent-rated	5.69 (2.97) 7.03 (2.45)	4.78 (2.90) 6.56 (2.36)	6.22 (2.89) 7.30 (2.47)	2.60 (2.67) 0.86 (2.00)	2.50 (2.29) 0.45 (0.83)	2.65 (2.83) 1.05 (2.32)	12,807.50*** 14,552.50***
Impairment with friends							
Self-rated Parent-rated	3.56 (2.94) 5.70 (2.47)	2.52 (2.58) 5.72 (2.62)	4.16 (2.97) 5.69 (2.39)	2.08 (2.13) 0.59 (1.44)	2.41 (2.42) 0.24 (0.58)	1.93 (1.99) 0.74 (1.67)	10,536.00*** 14,468.50***
Impairment at home							
Self-rated Parent-rated	4.99 (2.96) 4.75 (2.93)	4.50 (2.94) 4.85 (2.91)	5.27 (2.96) 4.70 (2.96)	2.23 (2.41) 0.63 (1.44)	2.28 (2.69) 0.48 (1.48)	2.21 (2.29) 0.69 (1.42)	12,425.00*** 13,739.50***

*ADHD* attention-deficit/hyperactivity disorder, *SD* standard deviation

*Note*: In the clinical group, *n* = 159 for the self-ratings and *n* = 162 for the parental ratings. In the reference group, *n* = 103 for the self-ratings and *n* = 94 for the parental ratings. Inattention and hyperactivity were assessed with the subscales of *Adult ADHD self-report scale for adolescents*, where each subscale ranges from 0 to 36. Emotional and conduct problems were assessed with the *Strengths & Difficulties Questionnaire*, where each subscale ranges from 0 to 10. Functional impairment was assessed with *Child Sheehan Disability Scale*, where overall impairment ranges from 0 to 30 and impairment in each life domain ranges from 0 to 10. Group differences were evaluated with the non-parametric Mann–Whitney U-test due to skewness in the reference group

^*^ *p* < .05

^***^ *p* < .001

### Associations between ADHD presentation and functional impairment

Mean differences in functional impairment between ADHD presentations are displayed in Table 2. ADHD-C was related to the highest levels of self-reported impairment in all life domains, whereas no significant differences were found between ADHD-I and ADHD UNS. The parental ratings showed that ADHD-C was related to more impairment in school than ADHD UNS, with no other differences observed between the ADHD presentations.

**Table 2:** Mean differences in functional impairment across ADHD presentations among adolescents with ADHD

	Self-ratings			Parental ratings		
Functional impairment	Mean (SD), n	F	Tukey’s	Mean (SD), n	F	Tukey’s
Overall impairment						
ADHD-C = a ADHD-I = b ADHD UNS = c	17.38 (6.59), 72 12.52 (7.37), 56 10.29 (5.81), 28	14.35***	a > b, c***	18.29 (6.32), 72 17.76 (6.19), 57 15.43 (5.54), 30	2.34	NS
Impairment in school						
ADHD-C = a ADHD-I = b ADHD UNS = c	6.75 (2.50), 72 5.34 (3.18), 56 4.00 (2.65), 28	10.75***	a > b* a > c***	7.38 (2.56), 72 7.25 (2.40), 57 6.07 (1.98), 30	3.34^*^	a > c^*^
Impairment with friends						
ADHD-C = a ADHD-I = b ADHD UNS = c	4.58 (2.93), 72 2.80 (2.91), 56 2.54 (2.27), 28	8.64***	a > b, c**	5.85 (2.44), 72 5.85 (2.49), 57 5.07 (2.55), 30	1.21	NS
Impairment at home						
ADHD-C = a ADHD-I = b ADHD UNS = c	6.04 (2.92), 72 4.38 (2.94), 56 3.75 (2.24), 28	9.04***	a > b, c**	5.07 (3.01), 72 4.67 (3.04), 57 4.30 (2.63), 30	0.78	NS

*ADHD* attention-deficit/hyperactivity disorder, *ADHD-C* combined presentation, *ADHD-I* predominantly inattentive presentation, *ADHD UNS* unspecified ADHD, *NS* non-significant,

*SD* standard deviation

*Note*. The statistical analyses were performed with one-way analysis of variance, with Tukey’s post -hoc test

Three participants were excluded from this analysis since they were categorized as manifesting with predominantly hyperactive/impulsive presentation

Functional impairment was assessed with *Child Sheehan Disability Scale*, where overall impairment ranges from 0 to 30 and impairment in each life domain ranges from 0 to 10

^*^ *p* < .05

^**^ *p* < .01

^***^ *p* < .001

### Associations between ADHD domains and functional impairment for adolescents with ADHD

#### Self-rated functional impairment

The results from the GLMs based on self-rated impairment are presented in Table 3. Both inattention and hyperactivity/impulsivity were associated with overall impairment and impairment with friends and at home, while only inattention was associated with impairment in school. When adjustments were made for conduct problems, emotional problems, medication status, and sex, the association with self-rated overall impairment remained statistically significant for inattention, but not for hyperactivity. However, the unique variance explained by inattention was attenuated from a large to a medium effect size. In addition, emotional problems were associated with overall impairment. Inattention remained associated with impairment in school, with a large effect size, while the association with impairment with friends was attenuated from a moderate to a small effect and the relationship to impairment at home was lost. Hyperactivity/impulsivity was still associated with impairment with friends, with a small effect size, whereas the association with impairment at home was no longer statistically significant. Emotional problems contributed to the explained variance of impairment in school and at home, while conduct problems were associated with self-rated impairment at home. Further, medication status was significantly associated with impairment with friends, suggesting that adolescents with medication had slightly higher ratings of impairment in this life domain.

**Table 3:** Associations between clinical characteristics and self-reported impairment among adolescents with ADHD

	Step 1 B (SE); η_p_^2^				Step 2 B (SE); η_p_^2^			
	Overall impairment	Impairment in school	Impairment with friends	Impairment at home	Overall impairment	Impairment in school	Impairment with friends	Impairment at home
Inattention	.53 (.09)***; .20	.26 (.04)***; .26	.14 (.04)***; .08	.12 (.04)**; .06	.40 (.10)***; .10	.22 (.04)***; .15	.11 (.05)*; .04	.08 (.05); .02
Hyperactivity/impulsivity	.20 (.07)**; .05	.00 (.03); .00	.09 (.03)**; .04	.11(.03)**; .06	.14 (.07); .02	.00 (.03); .00	.07 (0.3)*; .03	.07 (.03); .03
Conduct problems	–	–	–	–	.43 (.27); .02	.03 (.12); .00	.10 (.13); .00	.30 (.12)*; .04
Emotional problems	–	–	–	–	.80 (.21)***; .09	.33 (.09)***; .08	.17 (.10); .02	.31 (.10)**; .06
Female sex	–	–	–	–	− .86 (1.05); .00	-.63 (.45); .01	.51 (.48); .01	− .73 (.48); .02
ADHD medication	–	–	–	–	.94 (1.08); .00	-.02 (.47); .00	1.01 (.50)*; .03	− .05 (.50); .00

*ADHD* Attention-deficit/hyperactivity disorder, *SE* standard error

The statistical analyses were performed with general linear models conducted in two steps

Inattention and hyperactivity were assessed with the subscales of *Adult ADHD self-report scale for adolescents*, where each subscale ranges from 0 to 36. Emotional and conduct problems were assessed with the *Strengths & Difficulties Questionnaire*, where each subscale ranges from 0 to 10. Functional impairment was assessed with *Child Sheehan Disability Scale*, where overall impairment ranges from 0 to 30 and impairment in each life domain ranges from 0 to 10

*p < .05

**p < .01

***p < .001

#### Parent-rated functional impairment

The results from the GLMs based on parent-rated impairment are presented in Table 4. Inattention contributed to overall impairment and functional impairment in each life domain. In contrast, hyperactivity/impulsivity contributed significantly only to impairment at home. In the fully adjusted models, the association between inattention and overall impairment remained, with a large effect size. In addition, conduct problems and emotional problems contributed significantly to the explained variance of overall impairment. The associations between inattention and impairment in school and with friends remained significant, with a large and a moderate effect on these outcomes, respectively, whereas the association with impairment at home was attenuated to a small effect size. The association between hyperactivity/impulsivity and impairment at home was lost in the adjusted model. Emotional problems and conduct problems both contributed to the explained variance in impairment with friends, with conduct problems also found to be a strong associated predictor of impairment at home.

**Table 4:** Associations between clinical characteristics and parent-rated impairment in adolescents with ADHD

	Step 1 B (SE); η_p_^2^				Step 2 B (SE); η_p_^2^			
	Overall impairment	Impairment in school	Impairment with friends	Impairment at home	Overall impairment	Impairment in school	Impairment with friends	Impairment at home
Inattention	.57 (.08)***; .23	.27 (.03)***; .31	.16 (.04)***; .10	.14 (.04)**; .06	.50 (.08)***; .20	.27 (.04)***; .27	.13 (.04)**; .07	.10 (.04)*; .04
Hyperactivity/impulsivity	.09 (.06); .01	-.01 (.02); .00	.02 (.03); .00	.08 (03)*; .04	− .04 (.06); .00	-.02 (.03); .00	− .02 (.03); .00	.01 (.03); .00
Conduct problems	–	–	–	–	1.13 (.20)***;.17	.09 (.09); .01	.30 (.10)**; .06	.75 (.10)***; .25
Emotional problems	–	–	–	–	.42 (.17)*; .04	− .01 (.07); .00	.31 (.08)***; .09	.12 (.09); .01
Female sex	–	–	–	–	− .25 (.82); 00	.56 (.36); .02	-.61 (.39); .02	− .19 (.42); .00
ADHD medication	–	–	–	–	1.10 (.91); .01	.30 (.40); .00	.31 (.43); .00	.49 (.46); .01

*ADHD* Attention-deficit/hyperactivity disorder, *SE* standard error

*Note.* The statistical analyses were performed with general linear models conducted in two steps

Inattention and hyperactivity were assessed with the subscales of *Adult ADHD self-report scale for adolescents*, where each subscale ranges from 0 to 36. Emotional and conduct problems were assessed with the *Strengths & Difficulties Questionnaire*, where each subscale ranges from 0 to 10. Functional impairment was assessed with *Child Sheehan Disability Scale*, where overall impairment ranges from 0 to 30 and impairment in each life domain ranges from 0 to 10

*p < .05

**p < .01

***p < .001

## Discussion

In this cross-sectional study, we investigated the association between ADHD and the outcomes of functional impairment in several life domains during adolescence, as well as the influence of sex and comorbid symptoms on these outcomes. Functional impairment in daily activities was reported to be higher among adolescents with ADHD compared with peers without the diagnosis, and girls with ADHD rated more impairment than their male counterparts. Further, the combined ADHD presentation was associated with the highest levels of self-reported impairment, whereas parental ratings indicated comparable levels of overall impairment between the presentations. In the adjusted models, inattention was strongly associated with self- and parent-rated impairment in school, but hyperactivity/impulsivity was not, while both inattention and hyperactivity/impulsivity were modestly associated with self-rated impairment with friends. Both emotional problems and conduct problems contributed to the explained variance of self- and parent-rated impairment, and adjustment for these factors attenuated the effect of ADHD symptoms, especially in regard to impairment at home.

### Group and sex differences

In agreement with our first hypothesis, our results showed that adolescents with ADHD had higher levels of both self- and parent-rated impairment than peers without the diagnosis, which confirms earlier findings [10]. Given that a majority of participants in our clinical sample had ongoing pharmacological treatment for ADHD (76.5%), our results highlight the need for additional interventions in this age group, where impairment in daily activities should be specifically targeted. In our sample, girls with ADHD rated a higher degree of functional impairment than boys with ADHD, whereas no sex differences were found in the reference group. While this finding stands in contrast to those of studies using other informants, such as parents and teachers [27–29], it is in line with findings from previous studies among young adults, where female college students with ADHD rated more impairment than their male counterparts [30–32]. It has been hypothesized that males with ADHD might underestimate their impairment [30], which is a phenomenon that has been seen in younger boys with ADHD [46]. The descriptive statistics in our study also indicate that boys with ADHD rated less ADHD symptoms and emotional problems than girls with ADHD, which is in line with some previous findings [30, 47, 48]. It has been found that girls are diagnosed with ADHD at an older age than boys and the occurrence of a potential referral bias has been discussed, where females may need to present with more ADHD symptoms as well as psychiatric comorbidity in order for their ADHD to be identified [32, 49–51]. This may delay diagnostic assessment and access to ADHD treatment, and thus increase the risk of additional impairment [32], which is a topic that needs further investigation.

Since pharmacological treatment for ADHD was more common among the boys in our sample, it is possible that their lower rates of functional impairment reflected a perceived effect of medication. However, in line with previous research [27–29], no sex differences were observed in regard to parent-rated impairment. Hence, the influence of sex seems to vary depending on the informant. Corroborating this, we found low to moderate correlations between self- and parent-rated impairment, confirming the evidence of poor agreement between parents and youths [52, 53]. An adolescent’s own perceptions of impairment in daily life will likely affect his/her motivation and receptiveness for suggested interventions. Therefore, any potential discrepancy between parental and adolescent reports needs to be fully acknowledged and our results speak to the importance of taking both informants into consideration when planning and delivering interventions for this age group [5, 19, 52, 53].

### The influence of ADHD presentation on functional impairment

The results of the self-ratings confirmed our second hypothesis of ADHD-C being associated with the highest level of overall impairment, which is in agreement with previous findings [10]. This suggests that the number of fulfilled ADHD symptoms may affect the self-perceived level of functional impairment. In contrast, no differences were found between the three ADHD presentations in regard to parent-rated overall impairment – which was somewhat surprising given that ADHD UNS (including patients who had subthreshold symptoms at the time of the study) was one of the ADHD presentations. Still, recent findings indicate that ADHD is a disorder with symptoms that fluctuate over time, and functional impairment often persists even if symptom levels decrease [54]. Our hypothesis of comparable levels of impairment in school between ADHD-C and ADHD-I was supported only by parental ratings, which confirms earlier findings based on parental and teacher ratings [10]. Thus, the influence of ADHD presentation varied between informants. While ADHD-C seems to be perceived as more impairing by the adolescent him-/herself, the parental ratings suggest that functional impairment continues to be high even when a child presents with subthreshold symptoms.

### The influence of inattention and hyperactivity/impulsivity on functional impairment

When a more dimensional perspective on ADHD was used, the adjusted models revealed inattention as the strongest associated predictor of both self- and parent-rated impairment in school. This confirmed our third hypothesis, and is in line with previous findings [9–11]. In high school, lectures often become longer and students are expected to plan and organize their school work by themselves to a larger extent, meaning that attentional deficits might become a greater obstacle [17]. Accordingly, psychosocial treatments with a specific focus on improving academic and organizational skills [55, 56], which could decrease and/or compensate for attentional deficits, are warranted for this age group. In addition, parents and teachers need to be adequately supported in the implementation of interventions and environmental adaptions [57, 58].

Moreover, our third hypothesis of both ADHD domains being associated with impairment with friends and at home was partly confirmed, although the influence of hyperactivity/impulsivity was weaker than expected. In the first step of the GLM, both inattention and hyperactivity/impulsivity were associated with impairment with friends and at home, which is in agreement with earlier findings [10]. When adjusting for comorbid symptoms, sex, and medication status, the effect of the ADHD domains was partly attenuated in relation to impairment with friends and at home, which confirmed our fourth hypothesis. In the adjusted model, inattention was still associated with self- and parent-rated impairment with friends, as well as with parent-rated impairment at home, whereas hyperactivity/impulsivity was associated only with self-rated impairment with friends (with a small effect). These results suggest a relatively low influence of hyperactivity/impulsivity on daily impairment, which may be related to the age in this sample and the decrease of overt ADHD symptoms that is often seen in adolescence [1, 11, 12]. Still, symptoms of hyperactivity/impulsivity seem to have some influence on self-perceived social functioning. The lack of this association in the parental ratings might be due to the parents being less involved in adolescents’ interaction with peers [5], and that symptoms of hyperactivity/impulsivity tend to be more subtle in adolescents and therefore less observable [12, 59].

### The influence of comorbid symptoms on functional impairment

As mentioned above, when adjusting for co-existing psychiatric symptoms, the effects of the two ADHD domains were attenuated, which was in line with our fourth hypothesis and with previous findings [9]. Accordingly, comorbid symptoms seem to have a significant impact on functional impairment in adolescents with ADHD. Emotional problems were associated with self-rated overall impairment and impairment in school and at home, as well as with parent-rated overall impairment and impairment with friends. The combination of ADHD and internalized problems has previously been linked to overall impairment [25], academic impairment [26], and social impairment [5]. Conduct problems were only modestly associated with self-reported impairment at home, while their clear associations with parent-rated impairment with friends and at home were more in accordance with earlier findings [5, 9]. The associations between comorbid symptoms and impairment at home differed depending on the informants, which may be related to the fact that the phrasing of the question regarding this domain differed between the self-rating scale and the parental rating scale (see “Methods” Section). The results indicated that emotional problems were perceived as more impairing at home by the adolescents themselves, while conduct problems seemed to be more strongly predictive of impairment in joint family life. Further, internalized difficulties and the impact of these might be difficult for others to observe, meaning that self-reports of such problems may be of particular importance [52, 53, 60]. In contrast, youths have shown a tendency to underestimate their externalizing behavior and since these symptoms are often more apparent for the surroundings, inclusion of parental and/or teacher reports of overt symptoms has been recommended [60]. These results highlight the need of assessing and treating comorbid symptoms in adolescents with ADHD and emphasize the importance of using multiple sources of information, including self-reports.

## Limitations

This study is not without limitations. First, it was a cross-sectional study, precluding any conclusions regarding the potentially predictive value of the examined variables over time, and no causal relationships could be claimed on the basis of our findings. Second, we did not adjust for factors such as socioeconomic status (e.g., parental education level), cognitive ability, parental mental health, teen-parent relationship, and when the ADHD diagnosis was received, which leaves the question of potential confounders unanswered. Potential protective factors of functional impairment could also be explored further, since strengthening of these factors should be of particular interest in psychosocial interventions for ADHD [5, 61]. Third, even if factors associated with self-reported impairment were of specific interest in this study, the addition of more objective measures of functional impairment might have strengthened our conclusions. Fourth, the characteristics of the sample need to be considered. A majority of the participants in the clinical group were female (64%), which does not reflect the sex distribution of ADHD [1], and females were overrepresented in the reference group as well. Similar sex distributions have been found in previous Swedish studies on adolescent and adult psychiatric patients that involved diagnostic interviews, self-reports, and participating in group treatment [37, 62, 63]. This suggests a self-selection bias, where females in the Swedish cultural context may be more willing than males to participate in research projects, in particular those that require time and involvement from the participant him-/herself. Moreover, all participants in the clinical group had an ongoing contact with a CAP unit and completed their questionnaires as a pre-treatment measurement in the context of a clinical trial during the period 2015–2018. In contrast, the reference group members were recruited at their schools in 2019, without any subsequent intervention. Although both groups completed their questionnaires from home and no obvious societal changes occurred in the relevant time in Sweden, we cannot rule out that the different timing and context of the recruitment procedure affected their reports on impairment.

This study also has some strengths. To the best of our knowledge, this is one of few studies that has evaluated the association between ADHD and self- reported functional impairment for adolescents with ADHD. The use of multiple informants may have contributed to a more complete picture, while also highlighting the importance of accounting for the adolescents’ own perspectives. Investigating impairment across different life areas, rather than using one global measure of impairment, may have helped clarify the association between the examined predictor variables and the functional impairment in each life domain. Lastly, the inclusion of clinical patients recruited from several regions may arguably have strengthened the ecological validity of the study findings.

## Conclusions

Clarification of the associations between clinical characteristics and functional impairment is an important step in the development of interventions that effectively target functional impairment in adolescents with ADHD. This study replicated previous research findings suggesting that adolescents diagnosed with ADHD experience worse functioning across a broad range of important life domains, as compared with neurotypical peers. Findings from this study suggest that adolescent girls with ADHD may perceive more impairment than adolescent boys with the disorder, which needs be explored further. As regards self-ratings, the combined presentation of ADHD was found to be associated with the highest levels of functional impairment, indicating that the number of currently fulfilled ADHD symptoms could influence self-perceived impairment in daily life. Further, our study confirmed earlier evidence of inattention as a strong associated predictor of impairment in school, and indicated that interventions that can decrease symptoms of inattention and/or teach adolescents and their networks how to compensate for attentional deficits are warranted. Moreover, both emotional problems and conduct problems were significantly associated with impairment in several life domains, which highlights the need of assessing and treating comorbid symptoms in adolescents with ADHD. In addition, the poor to moderate agreement between self-ratings and parental ratings of functional impairment underscore the importance of using multiple informants, including the adolescents themselves, in both research and clinical practice with this group.

## Supplementary Information

**Additional file 1: Table S1. **Kruskal-Wallis H-test with pairwise comparisons of functional impairment across combinations of group and sex.

## Data Availability

Consent for sharing individual data outside the research team was not obtained. However, reasonable requests for patient-level data can be made to the principal investigator (JI) and will be considered after discussion with the ethical review board. Relevant data are included in the manuscript.

## Abbreviations

ADHD: Attention-deficit/hyperactivity disorder
ADHD-C: Combined presentation
ADHD-I: Predominantly inattentive presentation
ADHD: UNS unspecified ADHD
ASRS-A: Adult ADHD self-report scale for adolescents
CAP: Child and adolescent psychiatric
CSDS: Child Sheehan Disability Scale
GLM: General linear model
MINI-KID: Mini International Neuropsychiatric Interview for Children and Adolescents
ODD: Oppositional defiant disorder
SD: Standard deviation
SDQ: Strength and Difficulties Questionnaire
