# Supplementary material for: Associated predictors of functional impairment among adolescents with ADHD—a cross-sectional study
Source: Child Adolesc Psychiatry Ment Health. 2022 Apr 5;16:29. doi: 10.1186/s13034-022-00463-0 (PMC8985377; doi:10.1186/s13034-022-00463-0)
Supplement: Supplementary file 1 — Additional file 1: Table S1. Kruskal-Wallis H-test with pairwise comparisons of functional impairment across combinations of group and sex. [file 13034_2022_463_MOESM1_ESM.docx]

**Table S1** Kruskal-Wallis H-test with pairwise comparisons of functional impairment across combinations of group and sex.

|  | **Self-ratings** | | | **Parental ratings** | | |
| --- | --- | --- | --- | --- | --- | --- |
| **Functional impairment** | **Mean (SD)** | **H (df)** | **Pairwise group**  **comparisons** | **Mean (SD)** | **H (df)** | **Pairwise group comparisons** |
| **Overall impairment**  Boys with ADHD = a  Girls with ADHD = b  Boys reference group = c  Girls reference group = d | 11.79 (6.77)  15.64 (7.30)  7.19 (6.39)  6.79 (6.19) | 66.545 (3)^***^ | a > c ^**^, d^***^  b > a ^**^, c, d^***^ | 17.13 (6.13)  17.69 (6.21)  1.17 (2.44)  2.48 (4.94) | 157.615 (3)^***^ | a, b > c, d^***^ |
| **Impairment in school**  Boys with ADHD = a  Girls with ADHD = b  Boys reference group = c  Girls reference group = d | 4.78 (2.90)  6.22 (2.89)  2.50 (2.29)  2.65 (2.83) | 66.798 (3)^***^ | a, b > c, d^***^  b > a^**^ | 6.56 (2.36)  7.30 (2.47)  0.45 (0.83)  1.05 (2.32) | 153.802 (3)^***^ | a, b > c, d^***^ |
| **Impairment with friends**  Boys with ADHD = a  Girls with ADHD = b  Boys reference group = c  Girls reference group = d | 2.52 (2.58)  4.16 (2.97)  2.41 (2.42)  1.93 (1.99) | 28.187 (3)^***^ | b > a, d^***^, c^**^ | 5.72 (2.62)  5.69 (2.39)  0.24 (0.58)  0.74 (1.67) | 149.158 (3)^***^ | a, b > c, d^***^ |
| **Impairment at home**  Boys with ADHD = a  Girls with ADHD = b  Boys reference group = c  Girls reference group = d | 4.50 (2.94)  5.27 (2.96)  2.28 (2.69)  2.21 (2.29) | 52.998 (3)^***^ | a, b > c, d^***^ | 4.85 (2.91)  4.70 (2.96)  0.48 (1.48)  0.69 (1.42) | 120.260 (3)^***^ | a, b > c, d^***^ |

*ADHD* attention-deficit/hyperactivity disorder, *SD* standard deviation, *df* degrees of freedom

Note. Functional impairment was assessed with *Child Sheehan Disability Scale*, where overall impairment ranges from 0 to 30 and impairment in each life domain ranges from 0 to 10.

Non-parametric statistics were used due to skewness in the reference group.

** *p* < .01

*** *p* ≤ .001
